# Changes in Colorectal Carcinoma Genomes under Anti-EGFR Therapy Identified by Whole-Genome Plasma DNA Sequencing

**DOI:** 10.1371/journal.pgen.1004271

**Published:** 2014-03-27

**Authors:** Sumitra Mohan, Ellen Heitzer, Peter Ulz, Ingrid Lafer, Sigurd Lax, Martina Auer, Martin Pichler, Armin Gerger, Florian Eisner, Gerald Hoefler, Thomas Bauernhofer, Jochen B. Geigl, Michael R. Speicher

**Affiliations:** 1 Institute of Human Genetics, Medical University of Graz, Graz, Austria; 2 Department of Pathology, General Hospital Graz West, Graz, Austria; 3 Division of Oncology, Medical University of Graz, Graz, Austria; 4 Institute of Pathology, Medical University of Graz, Graz, Austria; University of Washington, United States of America

## Abstract

Monoclonal antibodies targeting the Epidermal Growth Factor Receptor (EGFR), such as cetuximab and panitumumab, have evolved to important therapeutic options in metastatic colorectal cancer (CRC). However, almost all patients with clinical response to anti-EGFR therapies show disease progression within a few months and little is known about mechanism and timing of resistance evolution. Here we analyzed plasma DNA from ten patients treated with anti-EGFR therapy by whole genome sequencing (plasma-Seq) and ultra-sensitive deep sequencing of genes associated with resistance to anti-EGFR treatment such as *KRAS*, *BRAF*, *PIK3CA*, and *EGFR*. Surprisingly, we observed that the development of resistance to anti-EGFR therapies was associated with acquired gains of *KRAS* in four patients (40%), which occurred either as novel focal amplifications (*n* = 3) or as high level polysomy of 12p (*n* = 1). In addition, we observed focal amplifications of other genes recently shown to be involved in acquired resistance to anti-EGFR therapies, such as *MET* (*n* = 2) and *ERBB2* (*n* = 1). Overrepresentation of the *EGFR* gene was associated with a good initial anti-EGFR efficacy. Overall, we identified predictive biomarkers associated with anti-EGFR efficacy in seven patients (70%), which correlated well with treatment response. In contrast, ultra-sensitive deep sequencing of *KRAS*, *BRAF*, *PIK3CA*, and *EGFR* did not reveal the occurrence of novel, acquired mutations. Thus, plasma-Seq enables the identification of novel mutant clones and may therefore facilitate early adjustments of therapies that may delay or prevent disease progression.

## Author Summary

Targeted therapies based on characteristics of the tumor genome are increasingly being offered to patients with cancer. For example, colorectal carcinomas that are wild type for *KRAS* are frequently treated with monoclonal antibodies targeting the Epidermal Growth Factor Receptor (EGFR). However, almost all patients with clinical response to anti-EGFR therapies develop resistance and underlying mechanisms are poorly understood. Because of the instability of tumor genomes the status of predictive biomarkers, such as the *KRAS* gene, can change during the course of disease. So-called “liquid biopsies”, e.g. analyses of circulating tumor DNA, provide genetic follow-up data non-invasively from peripheral blood. When using whole genome sequencing of plasma DNA (plasma-Seq) we observed that specific copy number changes of genes, such as *KRAS*, *MET*, or *ERBB2*, can be acquired under therapy and determine responsiveness to therapy. In fact, our data suggest that non-invasive genome profiling is capable of predicting responsiveness or emerging resistance to anti-EGFR therapy in the majority of cases. Hence, non-invasive testing of the current status of the tumor genome can help reduce of harm from erroneous therapeutic decisions and optimize treatment for maximal efficacy and minimal side effects, which is important for decreasing metastasized CRC-related morbidity and mortality.

## Introduction

Colorectal cancer (CRC) is an important and highly prevalent health problem and improvements in outcomes associated with novel targeted therapies could have important health impacts. To this end, molecular markers are increasingly being used for predictive and prognostic applications in CRC. For example, mutant *KRAS* is a predictor of resistance to treatment with monoclonal antibodies targeting the Epidermal Growth Factor Receptor (EGFR), such as cetuximab (Erbitux) [1], [2] or panitumumab (Vectibix) [3]. However, almost all patients with wild type *KRAS* and clinical response to anti-EGFR therapies develop acquired resistance within a few months of starting therapy [4], [5].

Other factors than *KRAS* mutation status likely affect response to anti-EGFR therapy, because the response rates among patients with wild-type *KRAS* are less than 20% [1], [6], [7]. Recent investigations have identified genes and proteins downstream of KRAS in the mitogen-activated protein kinase signaling pathway, which affect unresponsiveness to anti-EGFR therapy, including the *BRAF* V600E mutation, mutations in *NRAS* or *PIK3CA* (exons 9 and 20), or loss of PTEN or AKT expression [8]–[10]. Furthermore, several mechanisms of acquired (secondary) resistance to anti-EGFR therapies, such as expression of EGFR ligands [11], deregulation of the EGFR recycling process [12], amplifications of the genes *ERBB2* (also called *HER2*) [13], [14], *KRAS* [15], [16], and *MET* [17], have been identified. In addition, the EGFR ectodomain mutation S492R has recently been found to confer resistance to cetuximab [18]. On the other hand, several studies reported evidence that an increased *EGFR* copy number enhances response rates to anti-EGFR therapy [10], [19]–[21].

Hence, there is a growing number of markers predictive of response and survival in patients treated with anti-EGFR therapy. However, the evolution of these markers during disease course is unknown at present due to a lack of follow-up genetic data. To this end investigations are now increasingly employing blood-based assays that characterize cell-free DNA (cfDNA) in the plasma of patients with cancer [22]–[32]. Cancer cells can release tumor DNA into the circulation, which is frequently referred to as circulating tumor DNA (ctDNA) and ctDNA is a component of cfDNA [33], [34]. ctDNA can be used to deduce characteristics from the tumor genome non-invasively from a blood sample [33], [34]. For example, using the ctDNA in plasma the emergence of secondary *KRAS* mutations, which are responsible for acquired resistance in patients with CRC who had initially responded to cetuximab or panitumumab, has recently been reported [16], [35].

Using plasma-Seq we investigated whether genetic alterations associated with acquired resistance to anti-EGFR therapy can be identified by analysis of cfDNA. Plasma-Seq employs a benchtop high-throughput platform, i.e. Illuminas MiSeq instrument, and performs whole-genome sequencing from plasma at a shallow sequencing depth (i.e. 0.1–0.2×) to establish a genome-wide copy number profile of the tumor at low costs (<300€) within 2 days [32]. Thus, plasma-Seq allows an easy assessment about clonal evolution of the tumor genome. Furthermore, we performed highly sensitive deep sequencing for mutations in *KRAS* (exon 2), *PIK3CA* (exons 9 and 20), *BRAF* (V600E), and *EGFR* (S492R mutation in patients who received cetuximab).

## Results

We analyzed plasma samples from 10 patients with metastasized CRC (Table 1). In none of the primary tumors a *KRAS* mutation was detected and the patients received anti-EGFR treatment. In all patients we successfully conducted plasma-Seq and in addition, we performed targeted deep sequencing of genes associated with anti-EGFR resistance as outlined below.

**Table 1:** Patients' clinical characteristics.

Patient ID	Samples^1^	Sex	Age at diagnosis (in years)	Site of primary tumor	TNM classification		Site of metastases	Anti-EGFR treatment	Duration of Anti-EGFR Treatment
**#1**	**PT1, P1_1, P1_2, P1_3**	M	54	sigmoid rectum	pTx G2	biopsy only	liver	Panitumumab (Vectibix)	23 months
**#2**	**PT2, P2_1, P2_2, P2_3**	M	72	sigmoid colon	pT4a N1b (2/14) M1 G2	resection of primary	liver	Panitumumab (Vectibix)	6 months
**#3**	**PT3, LM3, P3_1**	F	70	transverse colon	pT3 N1 (2/40) M0 G2	no metastases at time of diagnosis, progression after 9 months	liver, bone, spleen	Cetuximab (Erbitux)	8 months
**#4**	**PT4, P4_1**	M	80	rectum	pT3 N1 M1 G2	biopsy only	liver (multiple lesions), local recurrence in rectum	Panitumumab (Vectibix)	8 months
**#5**	**PT5, P5_1**	M	65	rectum	pT3 N1 (1/14) M1 G2	resection of primary	liver	Panitumumab (Vectibix)	7 months
**#6**	**P6_1, P6_2**	M	62	splenic flexture	pT3 N2 (7/13) M1 G2	resection of primary	liver, peritoneal carcinomatosis, adrenal gland	Cetuximab (Erbitux)	9 months
**#7**	**P7_1**	M	81	splenic flexture	pTx M1	biopsy only	liver	Cetuximab (Erbitux) and Panitumumab (Vecitibix)	8 months
**#8**	**M8, P8_1, P8_2**	M	55	rectum	pT3 N2 (8/30) M1 G2 L1	resection of primary	liver	Panitumumab (Vectibix)	5 months
**#9**	**P9_1**	M	60	rectum	pTx G2	biopsy only	liver	Cetuximab (Erbitux)	30 months
**#10**	**P10_1, P10_2, P10_3**	F	65	sigmoid colon	pT3 N2 (4/30) M0 G2	no metastases at time of diagnosis, progression after 3 years	lymph nodes intraabdominal	Panitumumab (Vectibix)	6 months

1 Sample names starting with “PT” indicate those derived from primary tumors, with “M” from a metastatic lesion, “LM” from a liver metastasis, and “P” indicates the plasma samples.

### Targeted deep sequencing of EGFR resistance associated genes

We conducted targeted deep sequencing for the 7 most common *KRAS* mutations in codons 12 and 13 (i.e. G12R, G12D, G12C, G12A, G12S, G12V, G13D), the *BRAF* V600E mutation, exon 9 and 20 *PIK3CA* mutations, and for the *EGFR* S492R mutation in patients who received cetuximab (Table S1). In addition, we included plasma samples from our previous study [26] with known percentage of ctDNA reflected in *KRAS* mutations as positive controls. Plasma-Seq allows an estimation of tumor DNA fraction in the plasma [32], which was above 10% of total cfDNA in all samples. Hence, the detection limit of deep sequencing, which is in the range of 1% [26], was sufficient for mutation detection in our plasma samples. However, sequencing of the aforementioned genes revealed mutations only in exon 9 of the *PIK3CA* gene in the plasma of 3 patients (#2, #5, and #8) (Table S1). Such exon 9 *PIK3CA* mutations were discussed not to have an independent effect on anti-EGFR efficacy [8] and in all three patients we found the same mutation also in pretreatment samples, i.e. primary tumor (#2 and #5) or metastasis (#8) (Table S1) suggesting that targeted deep sequencing has not contributed to the identification of therapy related changes in the tumor genomes of our patient cohort.

### Plasma-Seq identifies CRC associated copy number changes

Altogether we analyzed 18 plasma samples from the 10 patients, the mean coverage for the entire genomes was 0.16× (Table S2). For comparison one representative plasma copy number profile from each patient and an example of a control, i.e. plasma-Seq from a male person without cancer, are shown in Figure S1.

All plasma samples from patients showed CRC associated copy number changes (www.progenetix.org; [36]), such as loss of the chromosomal 5q22 region harboring the *APC* (adenomatous polyposis coli) gene (*n* = 1; P3_1 in Figure S1), and loss of chromosome arms 17p (*n* = 5; P1_2, P2_1, P4_1, P7_1, and P9_1 in Figure S1), and 18q (*n* = 7; P2_1, P3_1, P4_1, P6_1, P7_1, P8_1, and P9_1 in Figure S1). Interestingly, we observed loss of 8p and gains of 8q and 20q, which are among the most commonly observed copy number changes in CRC (www.progenetix.org; [36]), in all patient derived plasma samples.

In order to determine whether the number of sequenced reads for an individual patient sample deviates from patterns in normal samples, we calculated z-scores. We and others [22], [32] had defined z-scores of <−3 and >3 as significantly under- and overrepresented, respectively. To this end we first calculated log2-ratios, which we used for segmentation to achieve regions with similar copy-number values [32]. These segments were then used for calculation of so-called “segmental z-scores” by comparing the respective log2-ratios with those from a cohort of individuals of the same sex but without cancer [32]. Using these z-score calculation criteria we determined genetic alterations at chromosomal levels, such as focal amplifications and chromosomal polysomies. Focal amplifications refer to high-level genomic gains of circumscribed genomic regions, often encompassing just one or a few genes. In contrast, chromosomal polysomies represent variable degrees of chromosomal gains and often affect larger chromosomal regions, i.e. chromosome arms or entire chromosomes. Losses of chromosomal regions were also determined based on segmental z-score calculations.

Focal amplifications of *KRAS* on chromosome 12p12.1 [15], [16], *MET* (7q31.2) [17], and *ERBB2* (17q12) [13], [14] have been shown to be associated with acquired resistance in tumors that do not develop *KRAS* mutations during anti-EGFR therapy. Furthermore, several studies reported evidence of a relationship between polysomies involving the *EGFR* gene (7p11.2) and anti-EGFR efficacy. Mean *EGFR* copy numbers in the range of 2.5–2.9/nucleus in ≥40% of analyzed cells were suggested as relevant cutoff points to discriminate between responders and non-responders to anti-EGFR therapy [19]–[21]. Hence, the main focus of our study was on the regions known to affect anti-EGFR treatment response, i.e. *KRAS*, *MET*, *ERBB2*, and *EGFR*. Details on read-counts, log2-ratios, z-scores, and relative copy numbers for the segments harboring the respective genes are listed in Table S3; a summary of findings is in Table S4.

### Acquired focal *KRAS* amplifications under anti-EGFR therapy

Plasma-Seq indeed allowed us to observe the emergence of novel copy number changes, which were closely associated with the development of clinical anti-EGFR resistance.

**Figure 1:**
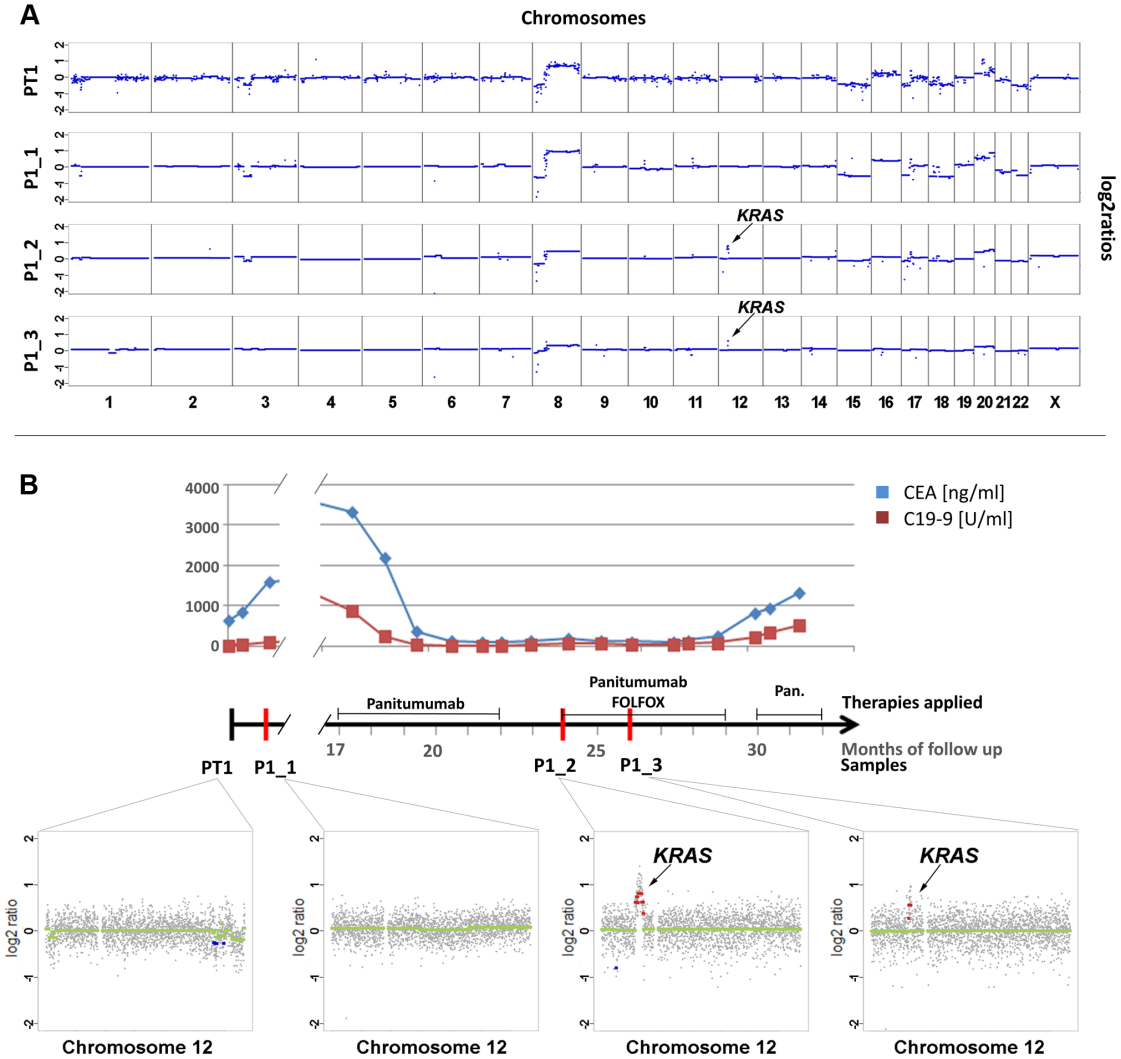
Emergence of *KRAS* amplification during panitumumab therapy in patient #1. (a) Copy number analyses of the primary tumor (PT1) and three plasma samples (annotated as P1_1, P1_2, P1_3). The dates of plasma sampling are shown in (b). The X- and Y-axes indicate the chromosome and the log2-ratios, respectively. The location of *KRAS* is indicated for plasma samples P1_2 and P1_3. (b) The units on the timeline in the center are in months, the dates of our blood collections are indicated by red bars. Above the time line the timing of anti-EGFR therapy and the CEA (ng/ml) and CA 19-9 (U/ml) levels (in blue and red lines, respectively) are illustrated. Below the timeline are zoomed in log2-ratio plots of chromosome 12 (PT1, month 0; P1_1, month 1; P1_2, month 24; and P1_3, month 26). Segments with identical log2-ratios whose log2-ratios is <0.2 are depicted with blue dots, segments with identical log2-ratios and log2-ratios >−0.2 in red dots, and segments with log2-ratios between −0.2 and 0.2 in green. (FOLFOX: FOL-Folinic acid (leucovorin) + F-Fluorouracil (5-FU) + OX-Oxaliplatin (Eloxatin); Pan.: Panitumumab).

When patient #1 was diagnosed he already had liver metastases and only biopsies from the primary tumor were obtained. Whole-genome sequencing of primary tumor DNA (PT1) revealed multiple of the aforementioned copy number changes frequently observed in colorectal cancer (Figure 1a). When we performed our 1^st^ plasma-Seq analysis one month later (P1_1) we observed, as expected, an almost identical pattern of copy number changes (Figure 1a). For the following 16 months the patient received various palliative treatments and was then switched to panitumumab monotherapy. Initially the patient responded with a marked decrease of tumor markers carcinoembryonic antigen (CEA) and carbohydrate antigen 19-9 (CA 19-9) (Figure 1b). However, after 7 months of treatment radiological progression was noted (new liver metastases). At this time we obtained our 2^nd^ blood sample (P1_2) and plasma-Seq revealed a similar pattern of copy number changes as in the previous analyses (Figure 1a). However, a novel focal amplification of the chromosomal region 12p12.1, harboring the *KRAS* gene was identified (Figure 1a, b). The segmental z-score for the 12p12.1 region was 23.9 corresponding to a high-level gain. For the next 4 months the patient was treated with FOLFOX (FOL-Folinic acid [leucovorin]; F-Fluorouracil [5-FU]; OX-Oxaliplatin [Eloxatin]) in addition to panitumumab, which resulted in stable disease (Figure 1b). During this time, we obtained our 3^rd^ blood sample (P1_3), which confirmed the presence of the *KRAS* amplification (Figure 1a, b). When a maintenance treatment with panitumumab monotherapy was tried the patient did not respond, but showed radiological progression and increase of tumor markers CEA and CA19-9 (Figure 1b). Another focal amplification on 17q11.2 (chr17:26,205,340–29,704,695) had already been present in both pretreatment samples (PT1) and (P1_1) and did not contain the *ERBB2* gene (exact position of *ERBB2*: chr17q12:37,844,167–37,886,679) (Figure S2), and is therefore likely unrelated to the development of anti-EGFR resistance.

**Figure 2:**
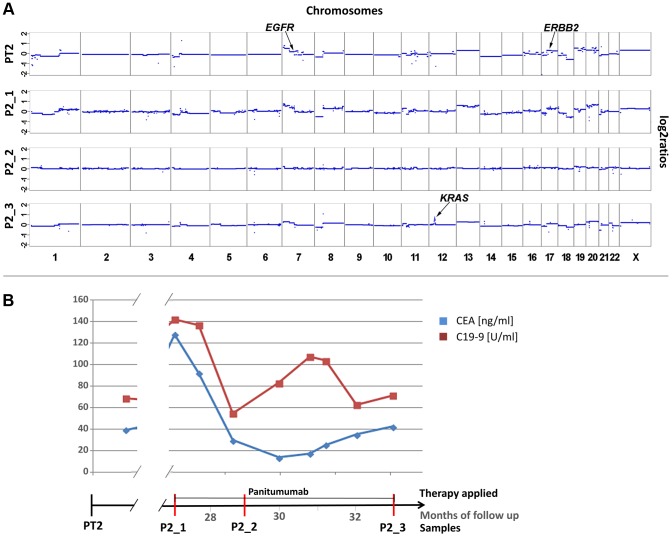
Appearance of *KRAS* amplification after 6 months of panitumumab therapy in patient #2. (a) Copy number analyses of the primary tumor (PT2) and three plasma analyses (P2_1, month 27; P2_2, month 29; and P2_3, month 33). The locations of the *EGFR*, *ERBB2* and *KRAS* genes are indicted in PT2 and P2_3, respectively. The dates of plasma sampling are shown in (b). (b) The timeline indicates the dates of our blood collections (red bars), the duration of the panitumumab therapy, and the respective CEA (ng/ml) and CA 19-9 (U/ml) (in blue and red, respectively) values.

For patient #2 we analyzed a first plasma sample after a disease course of 2 years and 3 months, immediately before his therapy was switched to panitumumab because of progressive disease. Despite the long time interval between initial diagnosis and our blood collection the copy number changes in the plasma sample showed a marked resemblance to those of the primary tumor (compare PT2 with P2_1 in Figure 2a). With respect to the above listed anti-EGFR therapy relevant regions we noted gains of 7p (*EGFR* z-scores: PT2: 7.79; P2_1: 10.9) and 17q (*ERBB2* z-scores: PT2: 6.69; P2_1: 5.6) in both samples (Figure 2a). The *EGFR* gain correlated excellently with an initial good response to anti-EGFR therapy as previously reported [19]–[21], because tumor markers CEA and CA19-9 decreased (Figure 2b) and when we repeated our plasma-Seq analysis two months later we observed an almost balanced copy number profile, indicating a very low ctDNA percentage (P2_2 in Figure 2a). However, another 4 months later, employing plasma-Seq, we identified a *KRAS* amplification (z-score *KRAS*: 10.59; P2_3 in Figure 2a) for the first time. This plasma-Seq result prompted a re-staging of the patient, which indeed revealed progressive disease.

### Acquired chromosome 12p polysomy and co-occurrence of *KRAS* and *MET* focal amplifications

**Figure 3:**
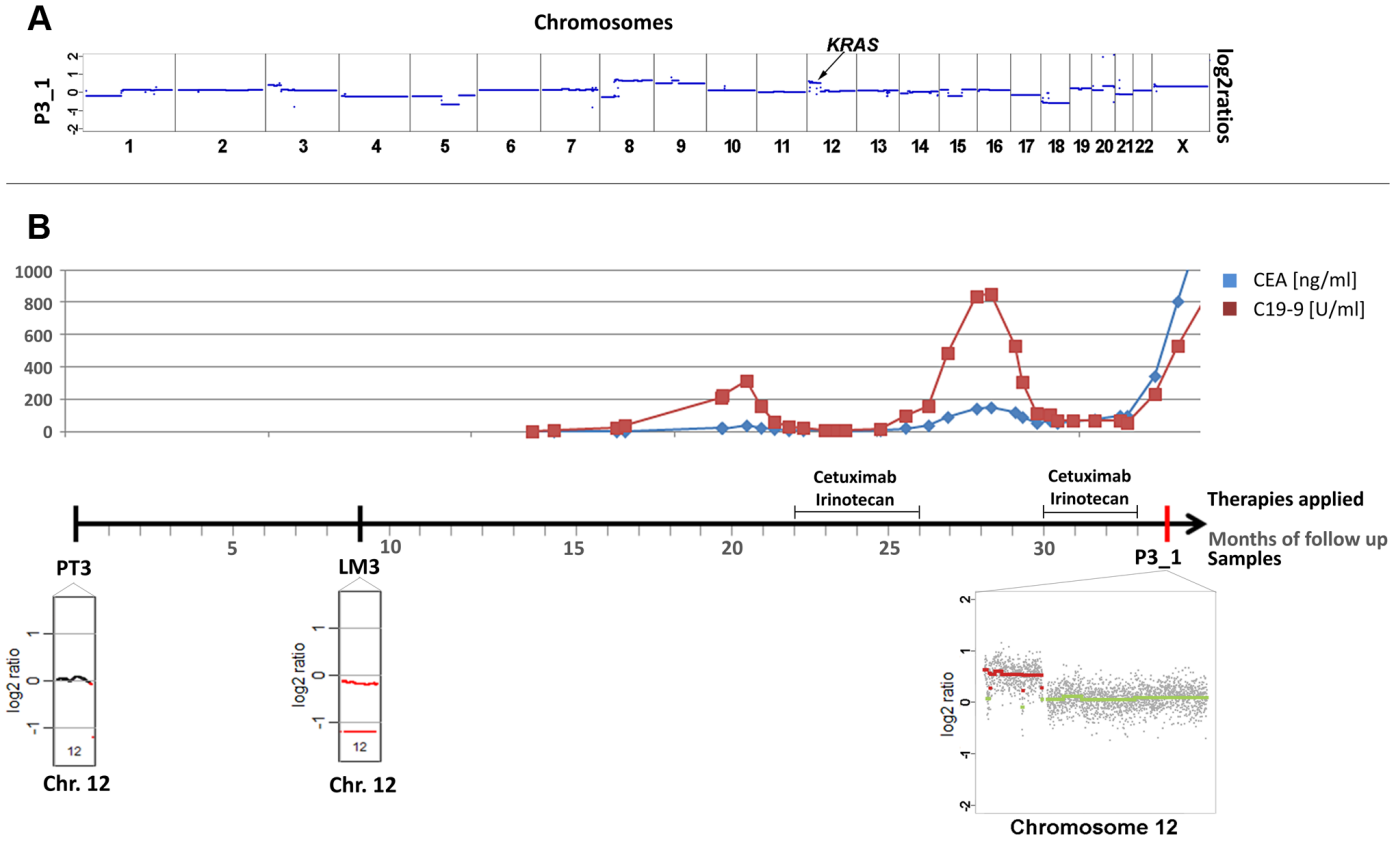
Occurrence of a chromosomal 12p polysomy under cetuximab therapy in patient #26C1. (a) Plasma-Seq (P3_1) confirmed the vast majority of the copy number changes observed in the two pre-treatment samples PT3 and LM3 (Complete array-CGH profiles of these two pre-treatment samples are shown in Figure S3). In plasma-Seq the high-level gain of the entire chromosome 12p including the *KRAS* gene was a novel change. (b) Time line indicating the timing of two cycles of cetuximab and CEA (ng/ml) and CA 19-9 (U/ml) (in blue and red, respectively) levels. Below the time line are chromosome 12 plots made by array-CGH [from the primary tumor (PT3), month 0; and a liver metastasis (LM3), month 9; black: balanced regions; red: lost regions] or by plasma-Seq (P3_1, month 35; color designation as in Figure 1).

Patient #3 received two cycles of cetuximab each for a 4 months period of time, which were administered 22 and 30 months, respectively, after diagnosis of the primary tumor (Figure 3). After the 2^nd^ cycle she developed resistance as documented by increasing CEA levels and radiographic progress (increasing size of abdominal metastases). As pre-treatment samples material from the primary tumor and of a liver metastasis, which had been resected 9 months after initial diagnosis, were available. Both primary tumor (PT3) and liver metastasis (LM3) shared many copy number changes as revealed by array-CGH (Figure S3); however, chromosome 12 was balanced in the primary tumor whereas it was lost in the liver metastasis (Figure 3; Figure S3). Our plasma-Seq analysis (P3_1), performed 35 months after the initial diagnosis, identified a novel high-level gain of the entire short arm of chromosome 12, which included the *KRAS* gene. A chromosome 12p z-score of 28.99 suggested that this chromosome arm was not only duplicated but present in multiple copies (Figure 3a).

**Figure 4:**
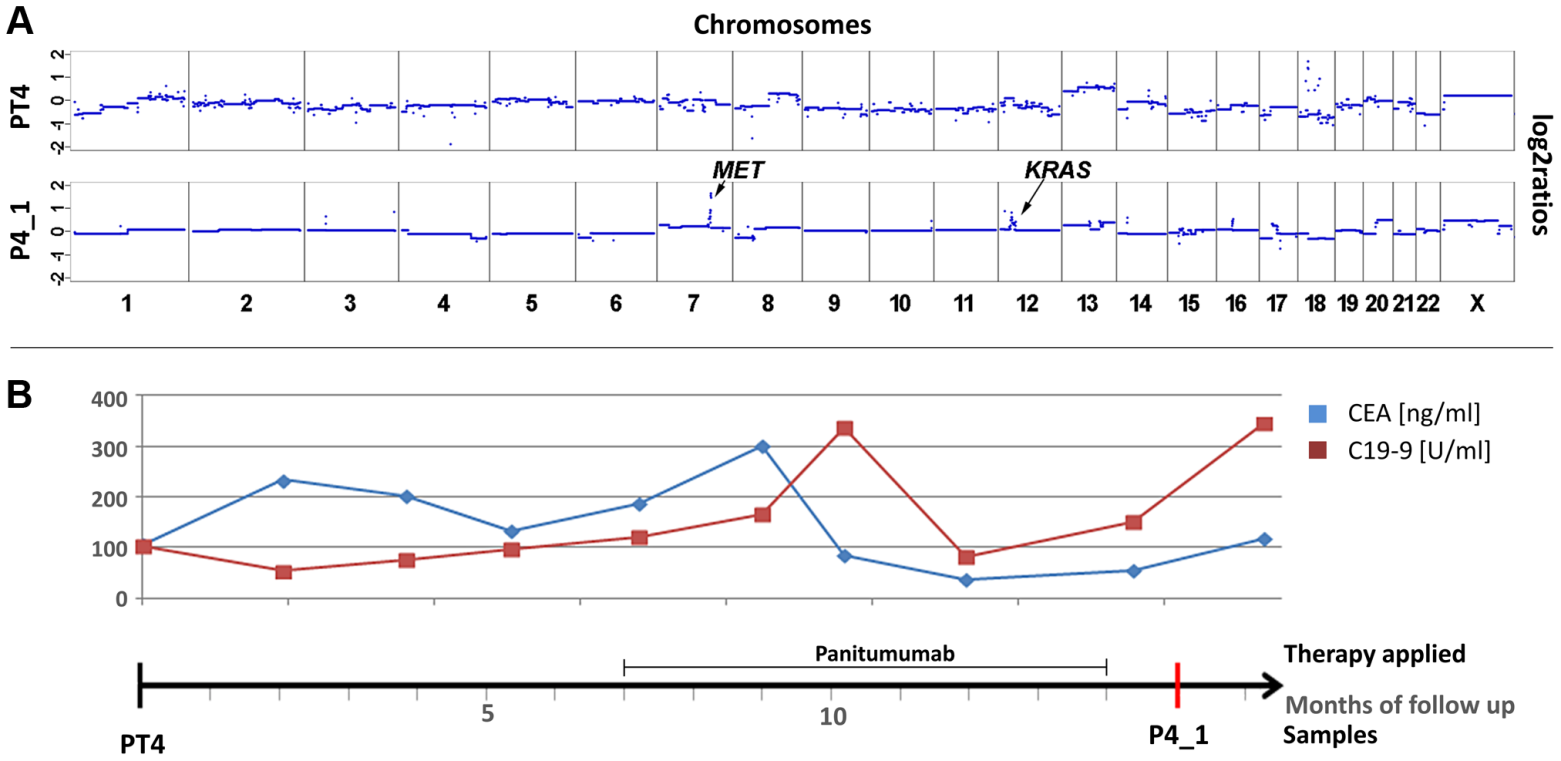
Co-occurrence of amplifications of the *KRAS* and *MET* genes observed after 7 months of treatment with panitumumab in patient #4. (a) Copy number profiles of the primary tumor (PT4) and the plasma DNA (P4_1) obtained 15 months later. The focal amplifications of the *MET* and *KRAS* genes on chromosomes 7 and 12, respectively, are annotated in the plasma sample. (b) The units in the time line correspond to months. Above the time line the period of panitumumab therapy and the CEA (ng/ml) and CA 19-9 (U/ml) (in blue and red, respectively) levels are illustrated.

We also observed the co-occurrence of a *KRAS* focal amplification with another focal amplification of an anti-EGFR therapy relevant gene, i.e. the *MET* gene. For patient #4 we tried to analyze the primary tumor (PT4) as a pre-treatment sample. However, as the primary tumor was inoperable, it was not resected but only biopsied so that only very little material was available. Hence, in this case it was not possible to tell whether the analyzed material was indeed representative for the primary tumor (Figure 4a). Patient #4 was treated with panitumumab and initially responded very well. However, after 5 months with anti-EGFR treatment increases of CEA and CA 19-9 were noted (Figure 4b). In a post-treatment plasma sample (P4_1) we observed two focal amplifications, again of 12p12.1 including *KRAS* and of 7q31.2 harboring the *MET* gene (Figure 4a). The respective z-scores were 13.63 for *KRAS* and 28.13 for *MET*. Furthermore, plasma-Seq revealed gains close to the centromeres of chromosomes 16 and 17. The focal amplicon on chromosome 16p11.2 (chr16:32,163,432–33,818,739) did not contain any gene previously implicated in anti-EGFR response, whereas the gain on chromosome 17 did not include the *ERBB2* gene (z-score: 2.54).

### Copy number changes in *MET*, *ERBB2*, and *EGFR*

**Figure 5:**
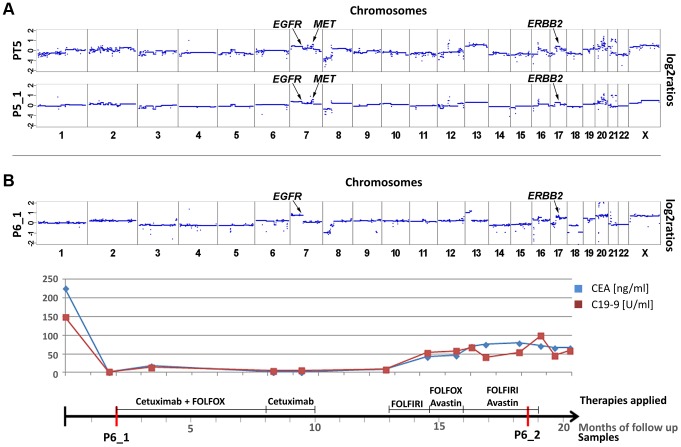
Cases with copy number changes in *EGFR*, *MET*, and *ERBB2*. (a) Copy number profiles of the primary tumor (PT5) and a plasma sample (P5_1) of patient #5, the time interval between both samples is almost 3 years. Both samples show a focal amplification of the *MET* gene and, furthermore regions on 7p and 17q, harboring the *EGFR* and the *ERBB2* genes, respectively, are gained. The focal amplification on chromosome 12q13.13-12q13.3 does not include the *KRAS* gene (for further details see text). (b) Plasma-Seq for patient #6 prior to anti-EGFR therapy with cetuximab (P6_1) showed several copy number changes, including gains of 7p (*EGFR*) and 17q (*ERBB2*). The time line indicates the low CEA and CA 19-9 levels during treatment with cetuximab. (FOLFOX: FOL-Folinic acid (leucovorin) + F-Fluorouracil (5-FU) + OX-Oxaliplatin (Eloxatin); FOLFIRI: FOL-Folinic acid (leucovorin) + F-Fluorouracil (5-FU) + IRI-irinotecan).

Although patient #5 received panitumumab and irinotecan for a period of six months, his liver metastases continued to progress. Copy number profiles of both the primary tumor (PT5) and a plasma sample (P5_1) obtained after panitumumab treatment had marked similarities (Figure 5a), although the time interval between the samples was 2 years and 11 months. In both samples we observed copy number changes in three regions which can affect anti-EGFR therapy: a focal amplification of the *MET* gene (z-scores PT5: 20.6; P5_1: 19.0), and polysomies of 7p (*EGFR* z-scores PT5: 12.0; P5_1: 10.5), and the 17q12 region (*ERBB2* z-scores: PT5: 5.7; P5_1: 5.0). Additional amplifications on other chromosomes, e.g. on chromosome 12q13.13-12q13.3 (chr12:51,639,133–56,882,181), which did not contain the *KRAS* gene on 12p12.1, were also present in both pre- and post-treatment samples. These amplicons did not contain genes which have yet been discussed within the context of affecting anti-EGFR therapies. As amplification of the *MET* gene has recently been shown to drive resistance to anti-EGFR therapies [17], this copy number change is the best candidate to explain the poor treatment response.

In patient #6 plasma-Seq (P6_1) revealed gains of 7p (*EGFR* z-score: 11.7) and of 17q (*ERBB2* z-score: 10.4) (Figure 5b) prior to cetuximab therapy, i.e. a similar copy number constellation as patient #2 prior to his therapy. This patient responded also very well to the treatment with remission of intrahepatic metastases and retroperitoneal lymph nodes and low CEA and CA 19-9 levels (Figure 5b) after treatment with cetuximab for 8 months. However, treatment had to be terminated because of cutaneous side effects and as several weeks later an increase of CEA and CA 19-9 levels was observed, and treatment had to be continued with chemotherapy (Figure 5b). We obtained a second blood sample during this time, but the ctDNA fraction was very low so that we did not obtain novel information.

### High-level focal amplification of *ERBB2* and poor treatment response

**Figure 6:**
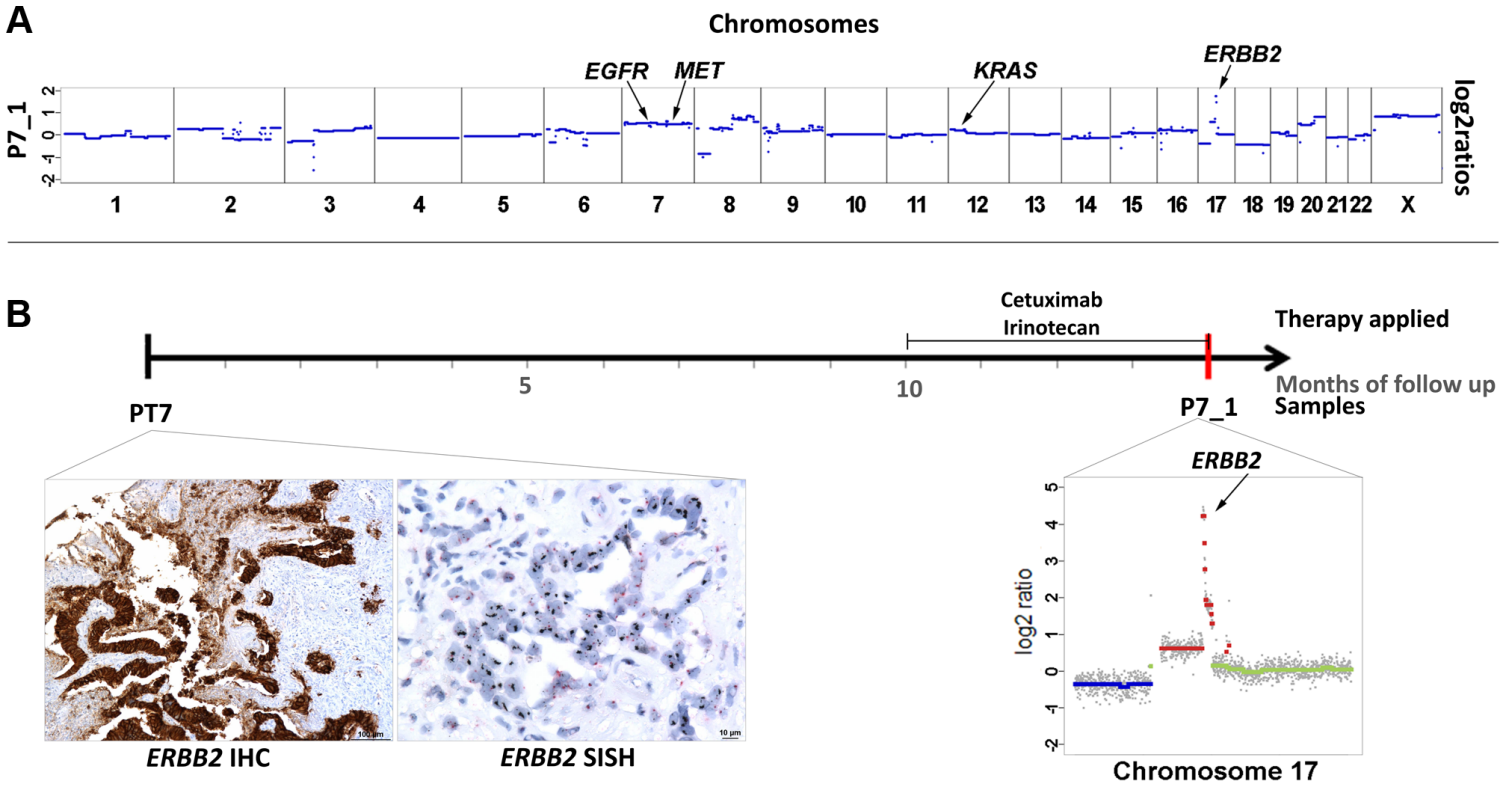
High-level focal amplification of *ERBB2* in patient #7 treated with cetuximab. (a) Copy number profile of the plasma sample (P7_1) and the locations of the *EGFR*, *MET*, *KRAS*, and *ERBB2* genes. (b) The time line illustrates the duration of cetuximab treatment and the date (red bar) of our blood collection. Below the time line are the ERBB2 immunohistochemistry and silver in situ hybridization (SISH) images of the biopsy material from the primary tumor (month 0) showing an immunoreactive score 3+ and high amplification, respectively. Her2/neu signals are black in the SISH technique. Furthermore, a plot of chromosome 17 from P7_1 obtained at month 14 is illustrated (note that the scale of the Y-axis reaches till 5 to illustrate the high-level gain; color designation as in Figure 1).

In patients #2 and #6 we had observed increased z-scores for both *EGFR* and *ERBB2*. In both cases there was an initial good response confirming previous reports that increased *EGFR* copy numbers enhance response rates to anti-EGFR therapy [19]–[21]. However, at the same time amplifications of *ERBB2* were reported to be associated with resistance [13], [14]. Hence, in these two cases the *EGFR* and not the *ERBB2* copy number appeared to have determined treatment outcome. However, another patient, i.e. #7, may contribute to the elucidation of the role of *ERBB2* in anti-EGFR therapy. This patient was treated with a combination of cetuximab and irinotecan after a disease course of 10 months (Figure 6). However, after only 3 months massive radiographic progress (increasing size of intrahepatic metastases and retroperitoneal lymph nodes) was noted. Plasma-Seq (P7_1) performed at this time revealed a focal high-level amplification of *ERBB2* with a z-score of 196.4. Furthermore, the short arm of chromosome 12 with the *KRAS* gene (z-score: 7.3) and the entire chromosome 7 (z-scores for *EGFR*: 17.5, and *MET*: 19.3) were also overrepresented (Figure 6a). The only available pre-treatment sample was a biopsy of the primary tumor and immunohistochemistry and SISH (silver in situ hybridization) revealed an extensive *ERBB2* immunoreactivity (immunoreactive score: 3+) and increased *ERBB2* signals with a highly increased Her2/CEP17 ratio (12.6) suggesting that the amplification had already been present at the time of initial diagnosis (Figure 6b). Based on previous reports [13], [14] the high-level *ERBB2* amplification may have been the main driver for the primary resistance to cetuximab although additional contributions by *KRAS* and *MET* are possible.

### Plasma-Seq does not reveal predictive markers in all cases

In 3 cases plasma-Seq did not reveal candidate regions as possible explanations for treatment response. In patient #8 we had as pretreatment samples a metastasis (M8) resected 5 months and a plasma sample (P8_1) obtained 45 months after initial diagnosis. Despite this long time interval copy number profiles of both samples were very similar (Figure S4). He had only a brief response to panitumumab treatment and relapsed within 4 months of treatment (radiological progression and increasing tumor markers). A post-treatment blood sample (P8_2) obtained 10 months later demonstrated again an almost identical copy number profile despite the progressive disease [P8_1 (z-scores: *EGFR*: 8.71; *MET*: 7.65), P8_2 (z-scores: *EGFR*: 4.73; *MET*: 4.73)] (Figure S4). As deep sequencing had also not revealed a novel mutation, we could not find a good explanation for the resistance towards panitumumab in this case. We observed a focal amplification on 16q12.1 (Chr16:51,265,518–52,958,468; z-score: 19.299), which was not present in the metastasis, in both plasma samples. As this amplicon had already been present in one of the two pre-treatment samples, i.e. P8_1, it was not acquired during anti-EGFR therapy. However, this amplicon represents an example that plasma-Seq identifies novel changes during tumor evolution, which may warrant further investigation.

Furthermore, we observed low ctDNA allele fractions which resulted in relatively unremarkable copy number plots in two patients. Patient #9 responded to cetuximab for more than 2 ½ years (Figure S5). After this long period of time a mild progress and mild increase of CEA and CA 19-9 were noted. Patient #10 received treatment with panitumumab after a disease course of 10 years, yet she did not respond well. In both cases plasma-Seq analysis showed only few copy number changes reflecting a low ctDNA fraction, in patient #10 even in three serial analyses (an exemplary profile is shown as P10_3 in Figure S1).

### Plasma-Seq verification by targeted copy number assays

As plasma-Seq is a new technique we verified the results for the genes *KRAS*, *MET*, and *ERBB2* by quantitative real-time PCR (RT-PCR). The quantitative RT-PCR results reflect relative but not real copy numbers because the fraction of ctDNA within the cfDNA varies and thus modulates the copy number. Nevertheless, the relative copy numbers of *KRAS*, *MET*, and *ERBB2* showed a close correlation to the respective log2-ratios (r^2^ = 0.686) (Figure S6). Furthermore, we also observed a close correlation between the quantitative RT-PCR relative copy numbers and the segmental z-scores (r^2^ = 0.557) (Figure S6) demonstrating that plasma-Seq reliably detects copy number changes.

## Discussion

Colorectal carcinomas that are wild type for *KRAS* are often sensitive to EGFR blockade [1], [3] and therefore *KRAS* testing can prevent both ineffective treatment and treatment-associated toxicity. However, to define CRC as *KRAS* mutant versus *KRAS* wild-type underestimates additional heterogeneity and calls for the identification of novel biomarkers for truly personalized medicine. Indeed, additional factors affecting anti-EGFR treatment have recently been identified, many of which are genes and proteins downstream of KRAS in the mitogen-activated protein kinase signaling pathway, such as *BRAF*, *NRAS*, *PIK3CA*, PTEN and AKT [8]–[12]. Furthermore, other *RAS* mutations than *KRAS* exon 2 mutations have recently been discussed as predictive markers [37].

However, despite this progress, the EGAPP (Evaluation of Genomic Applications in Practice and Prevention) Working Group (www.egappreviews.org) found only adequate evidence for an association of *KRAS* genotype at codons 12 and 13 with diminished treatment response to anti-EGFR therapy, but not for *BRAF* V600E, mutations in *NRAS* or *PIK3CA*, or loss of PTEN or AKT expression [6]. One reason for this uncertainty is the lack of genetic follow-up data in clinical studies. Often therapies are administered months or years after initial diagnosis and due to the instability of tumor genomes the status of predictive biomarkers obtained from the primary tumor may have changed over time. Recent progress in plasma DNA and CTC analyses now allows the monitoring of tumor genomes by non-invasive means [34], [38], [39]. Here we used plasma-Seq [32] and demonstrated that genetic follow-up data may include novel, acquired copy number changes, such as focal amplifications and chromosomal polysomies, which likely affect response to anti-EGFR therapy.

Regarding *KRAS* we made several interesting observations. Recently, it has been shown that the presence of *KRAS* amplification directly affects response to EGFR targeted agents and that *KRAS* amplification is a mechanism of resistance to EGFR targeted therapies in CRC [15], [16]. Currently, the frequency of *KRAS* amplifications is unknown. In colorectal cancer specimen *KRAS* amplifications were observed in only 0.67% (7/1039) [15] or 2.1% (2/96) [40] of tumors. In plasma samples from patients who progressed on cetuximab acquisition of *KRAS* mutations were a frequent finding [16], [35], whereas *KRAS* amplification was only observed in one patient [16]. However, these studies used 454 deep sequencing [16] or a digital ligation assay [35], i.e. methods with a high sensitivity for the detection of mutations but unsuitable to establish copy number levels. These differences in methods may explain our surprising and unexpected finding of acquired *KRAS* copy number changes in 4 of 10 (40%) patients. Although it will have to be further validated in larger cohorts an intriguing finding of our study is that *KRAS* amplifications as response to anti-EGFR therapy may be more prevalent than currently thought. Interestingly, in patient #1 the *KRAS* amplification was detected in plasma DNA at a time when CEA and CA19-9 levels were still at background levels. Similarly, in patient #2 the detection of the *KRAS* amplification was the reason for the initiation of a re-staging, which then showed clear evidence that the patient was indeed progressive. These two cases exemplarily demonstrate the potential power of plasma-Seq.

However, a potential shortcoming of our study is that we had no access to post-treatment tissues to confirm the plasma-Seq results. At the same time there were several lines of evidence supporting our interpretations: 1. We observed a very close correlation between the emergence of *KRAS* amplifications and the respective clinical course. 2. As mentioned before, *KRAS* amplifications are a recently established, novel mechanism of resistance against anti-EGFR therapy [15], [16]. 3. The *KRAS* amplification was the only new, acquired copy number change in plasma-Seq and we excluded genetic alterations in other oncogenes known to modulate EGFR signaling, such as mutations in *BRAF* [41], *PIK3CA* [8], or *EGFR* [18], [42] and *ERBB2* amplification [13], [14], which are established key determinants of resistance to anti-EGFR therapies. 4. We confirmed copy number changes observed with plasma-Seq by another method, i.e. with quantitative RT-PCR.

Recently it has been suggested that the development of resistance to *EGFR* blockade might be caused by rare cells with *KRAS* mutations that preexist at low levels in tumors with ostensibly wild-type *KRAS* genes [35]. Given that previous studies [15], [40] did not find *KRAS* amplifications in primary tumors in relevant numbers, it will be interesting to extend the analyses of primary tumors with more sensitive methods to identify the possible presence of such amplifications at the subclonal level.

Furthermore, our data confirm previous suggestions [15] that *KRAS* mutations and amplifications occur mutually exclusively. However, we also made the novel observation that *KRAS* amplification may co-occur with other anti-EGFR associated amplifications, such as the *MET* gene. Although the prevalence of *MET* amplification in untreated metastatic colorectal cancer was also reported to be low [17], [43]–[45], we identified two of them in 10 patients. It was shown that anti-EGFR therapies may select *MET*-amplified preexisting clones, which may then limit the efficacy of anti-EGFR therapies [17] and this may have been the mechanism of resistance in patient #4.

Activation of ERBB2 signaling, e.g. through *ERBB2* amplification, leads to persistent ERK 1/2 signaling, which was shown to be the principle mechanism of both primary and secondary resistance to cetuximab-based therapy in colorectal cancer patients [13], [14]. This may be the reason why patient #7 with a high-level focal amplification did not benefit from treatment with cetuximab. The level of *ERBB2* overrepresentation may determine treatment response, as lower gains in other cases did apparently not affect treatment response. Importantly, the high-level *ERBB2* amplification was only noted through our plasma-Seq analysis, as from the primary tumor only small samples from bioptic procedures insufficient for a detailed analysis of the tumor genome were available. Plasma-Seq initiated reanalysis of the remaining bioptic material by immunohistochemistry and FISH, which confirmed the *ERBB2* amplification. This suggests that in cases where only limited tumor material is available, a “base-line” plasma-Seq profile established at the time of initial diagnosis may help to guide therapeutic decisions.

Plasma-Seq may also contribute to the identification of patients who will likely benefit from anti-EGFR therapy. For example, several studies reported evidence of a relationship between increased *EGFR* copy number and anti-EGFR efficacy [19]–[21]. Indeed, patients with 7p polysomies were initially good responders. In fact, an interesting observation of our study is that copy number analyses alone were very powerful to detect associated mechanisms relevant for anti-EGFR treatment. In contrast, mutation analyses did not identify novel acquired mutations in genes previously associated with anti-EGFR resistance, such as *KRAS* [16], [35], *BRAF* [41], *PIK3CA* [8], or *EGFR* [18], [42].

A further potential limitation of our approach is that we cannot assess all possible mechanisms, which can affect response to anti-EGFR therapy. For example, decreased *PTEN* expression can be the result of mutations, allelic loss, and hypermethylation of the *PTEN* promoter region [10], [46], [47]. Our approach would miss epigenetic alterations, such as hypermethylation of promoters, and furthermore it cannot establish expression levels of genes. In this study we focused mainly on regions known to affect anti-EGFR treatment, i.e. *EGFR*, *MET*, *KRAS*, and *ERBB2*. However, our genome-wide approach should also allow mapping of novel regions which may be involved in anti-EGFR resistance. However, this will require larger cohorts and could not be achieved with the limited number of samples used in this study.

We demonstrated here that the status of predictive anti-EGFR markers may change in tumor genomes of patients with metastatic CRC. Thus, treatment decisions should not depend on the marker status of the primary tumor but on the current status as established by liquid biopsies [33], [34]. Hence, prospective clinical trials need to include evaluation of drug resistance mechanisms at the time of disease progression, which can now be achieved by non-invasive means. At present such trials should analyze post-treatment samples, i.e. tissue samples, in parallel to the liquid biopsies whenever possible to establish which approach yields the more representative result. Plasma-Seq is of particular utility in metastatic disease, i.e. the target population for nearly all early phase clinical trials. To this end, plasma-Seq represents an easy, fast, and affordable tool to provide the urgently needed genetic follow-up data in clinical studies. Hence, plasma-Seq may contribute to the identification of novel determinants of therapeutic response and may enable the early initiation of combination therapies that may delay or prevent disease progression.

## Materials and Methods

### Ethics statement

The study was approved by the Ethics Committee of the Medical University of Graz (approval number 21-229 ex 09/10), conducted according to the Declaration of Helsinki, and written informed consent was obtained from all patients.

### Patient population

The clinical data is summarized in Table 1. All patients had metastatic CRC and were seen in the Department of Internal Medicine, Division of Oncology, at the Medical University of Graz.

We could isolate DNA from pre-treatment tumor specimens in 5 patients where tumor tissue was available through surgical or bioptic procedures. In one patient immunohistochemistry (4B5 antibody, Ventana) and silver in situ hybridization (SISH, Ventana) were performed on biopsy material to establish the Her2/neu status before treatment (using a Benchmark Ultra platform). Partial data regarding copy number profiles made by array-CGH from patients #1 (P1_1), #7 (P7_1), #3 (P3_1), #6 (P6_1) were described in our previous papers [26], [48]. All plasma-Seq or whole-genome sequencing analyses and all mutation analyses presented here have not been previously published.

### Array-CGH

Array-CGH was carried out as previously described [26] using a genome-wide oligonucleotide microarray platform (Human genome CGH 60K microarray kit, Agilent Technologies, Santa Clara, CA, USA), following the manufacturer's instructions (protocol version 6.0).

### Plasma-Seq: Whole-genome sequencing (primary tumor; metastasis)

The methods were described in detail previously [26], [32]. In brief, plasma DNA was prepared using the QIAamp DNA Blood Mini Kit (Qiagen, Hilden, Germany). Shotgun libraries were prepared using the TruSeq DNA LT Sample preparation Kit (Illumina, San Diego, CA, USA) following the manufacturer's instructions with some exceptions: we used 5–10 ng of input DNA, we omitted the fragmentation step since plasma DNA has an enrichment of fragments in the range of 160 to 340 bp, for selective amplification of the library fragments that have adapter molecules on both ends we used 20–25 PCR cycles. The libraries were sequenced on an Illumina MiSeq (Illumina, San Diego, CA, USA). On the MiSeq instrument the run was initiated for 1×150 bases of SBS sequencing, including on-board clustering. On the completion of the run data were base called, demultiplexed on the instrument (provided as Illumina FASTQ 1.8 files, Phred+33 encoding), and FASTQ format files in Illumina 1.8 format were used for downstream analysis.

### Deep sequencing

Deep sequencing was performed with the Illumina MiSeq as described [48]. In brief, target specific primers were designed for all mutations as listed in Table S1 and Illumina specific adapters were attached to the 5′ ends in a second PCR run. Obtained sequence reads were base called using the Illumina MiSeq Reporter Software. Subsequently, reads were aligned to the human hg19 genome using Burrows-Wheeler Alignment (BWA, MEM-algorithm) [49] and alignments with mapping quality <15 were filtered. Bases sequenced with Phred-scores lower than 20 were masked in the alignment using an in-house script and mutations were visualized using Integrative Genomics Viewer (IGV) [50]. We set the threshold for reliable detection of a sequence variation at 1%, allelic fractions of <1% were considered as sequencing errors.

### Bioinformatics

We masked the pseudoautosomal region (PAR) of the hg19 genome and divided it into 50,000 windows, each containing the same amount of mappable reads.

Low-coverage whole-genome sequencing reads were mapped to the PAR-masked hg19 genome and reads in each window were counted and normalized by the median read-count obtained for each sample. We further normalized read counts according to the GC-content of each genomic window using LOWESS-statistics. In order to avoid position effects we normalized the sequencing data with GC-normalized read counts of plasma DNA of controls without malignant disease and calculated log2-ratios as detailed in [32]. Subsequently, we generated segments of similar copy-number values by applying both, circular binary segmentation (CBS) [51] and Gain and Loss Analysis of DNA (GLAD) [52] reflecting a summary of both algorithms as provided by the R-package CGHWeb [53], which generate mean log2-ratios for each identified segment.

The log2-ratio threshold for plotting of copy numbers was set to 0.2 for gains and −0.2 for losses.

Owing to variable ratios of tumor specific and normal alleles in plasma, accurate copy number calculations are not applicable. Furthermore, log2-ratios only indicate the relative copy number changes and are less sensitive at low allele fractions of tumor-specific DNA. For this reasons we applied z-score statistics. Z-scores indicate whether a region is gained or lost at a significant level (i.e. z-score >3 or <−3) compared to controls, i.e. individuals without cancer. Z-Scores for each segment were calculated by subtracting the mean GC-corrected read count of controls and dividing by standard-deviation of controls.

In order to check for the copy-number status of genes we calculated gene-specific z-scores (a z-score applied to test whether the copy number of a specific gene, i.e. ERBB2, significantly deviates from the control samples). Therefore, the chromosomal region of a specific gene was defined as a window and z-scores were calculated as described above [32]. However, for genes <100 kb in length gene-specific z-scores are not applicable since the standard deviation might be too large owing to the shallow sequencing depth. Hence, since most analyzed genes (except *BRAF* and *MET*) were <100 kb in length we used z-scores of the called segments where the genes are located.

### TaqMan Copy Number Assays

We used TaqMan Copy Number Assays from Life Technologies, Carlsbad CA, USA, to validate the copy number status of *KRAS*, *MET* and *ERBB2*. The respective assays are commercially available under the IDs Hs0239788_cn, Hs04993403, and Hs00450668_cn, respectively. We ran the TaqMan Copy Number Assays simultaneously with a TaqMan Copy Number Reference Assay (*hTERT*) in a duplex real-time polymerase chain reaction (PCR). The target assay contained two primers and a FAM dye labeled MGB probe and the reference assay contained two primers and a VIC dye-labeled TAMRA probe. PCR setup was performed according to the manufacturer's recommendations with the following exceptions: we increased the number of PCR cycles to 45 and decreased the amount of input DNA to 1–1.5 ng. The number of copies of the target sequence was determined by relative quantitation (RQ) using the comparative CT (ΔΔCT) method. Post-PCR data analysis of copy number quantitation experiments was done with Applied Biosystems CopyCaller Software.

### Accession numbers

All sequencing raw data were deposited at the European Genome-phenome Archive (EGA, http://www.ebi.ac.uk/ega/), which is hosted by the EBI, under the accession number EGAS00001000582.

## Supporting Information

Figure S1 Plasma-Seq copy number profiles of a control, i.e. from a male person without cancer (shown on top; non-cancer control), and one representative copy number profile from each patient (the left column shows the patient id, the right column the sample id). The X- and Y-axes indicate the chromosome and the log2-ratios, respectively.
(TIF)

Figure S2 Log2-ratio blots of chromosome 17 from PT1, P1_1, and P1_2, demonstrating a focal amplification close to the centromere on 17q11.2 (chr17:26,205,340–29,704,695). The localization of the *ERBB2* gene (chr17q12:37,844,167–37,886,679) is indicated by the black line.
(TIF)

Figure S3 Two pre-treatment samples, i.e. primary tumor (PT3) and a liver metastasis (LM3), analyzed by array-CGH shared many copy number changes with the exception of chromosome 12, which was balanced in the primary but lost in the metastasis (red: lost regions; green: gained regions; black: balanced regions).
(TIF)

Figure S4 Analyses of three different samples from patient #8: Plasma-Seq profiles of a metastasis (M8) obtained 5 months after initial diagnosis, and pre-and post-treatment plasma-samples (P8_1 and P8_2, respectively). He had a brief response to panitumumab treatment, but relapsed within 4 months of treatment (radiological progression and increasing tumor markers). A second blood sample 10 months (P8_2) later demonstrated an almost identical copy number profile despite the 10 months' time interval between the 1^st^ and the 2^nd^ sample and despite the progressive disease (for further details see text).
(TIF)

Figure S5 Patient #9 responded to cetuximab for more than 2 ½ years. After this long period of time a mild progress and mild increase of CEA and CA 19-9 were noted (CEA in blue and CA 19-9 in red). Plasma-Seq (P9_1) identified only few copy number changes, consistent with a low ctDNA fraction. (FOLFIRI: FOL-Folinic acid (leucovorin) + F-Fluorouracil (5-FU) + IRI-irinotecan).
(TIF)

Figure S6 Validation of the *KRAS*, *MET*, and *ERBB2* results established by plasma-Seq with TaqMan Copy Number assays. The graphs illustrate on the X-axes the relative copy numbers as established by the TaqMan assays and the Y-axes indicate the log2-ratios and the z-scores, respectively.
(TIF)

Table S1 Results of deep sequencing: The columns display the sample numbers and results for *KRAS* (codons 12 and 13), the *BRAF* V600E mutation, *PIK3CA* (exon-9 and exon-20), and for the *EGFR* S492R mutation.
(DOCX)

Table S2 Genome-wide coverage of plasma and tumor samples after plasma-Seq.
(DOCX)

Table S3 Details of copy-number aberrant segments detected by plasma-Seq.
(DOCX)

Table S4 Summary of copy number changes of *KRAS*, *EGFR*, *ERBB2*, and *MET*.
(DOCX)
