# Supplementary figures and images for: Changes in Colorectal Carcinoma Genomes under Anti-EGFR Therapy Identified by Whole-Genome Plasma DNA Sequencing
Source: PLoS Genet. 2014 Mar 27;10(3):e1004271. doi: 10.1371/journal.pgen.1004271 (PMC3967949; doi:10.1371/journal.pgen.1004271)

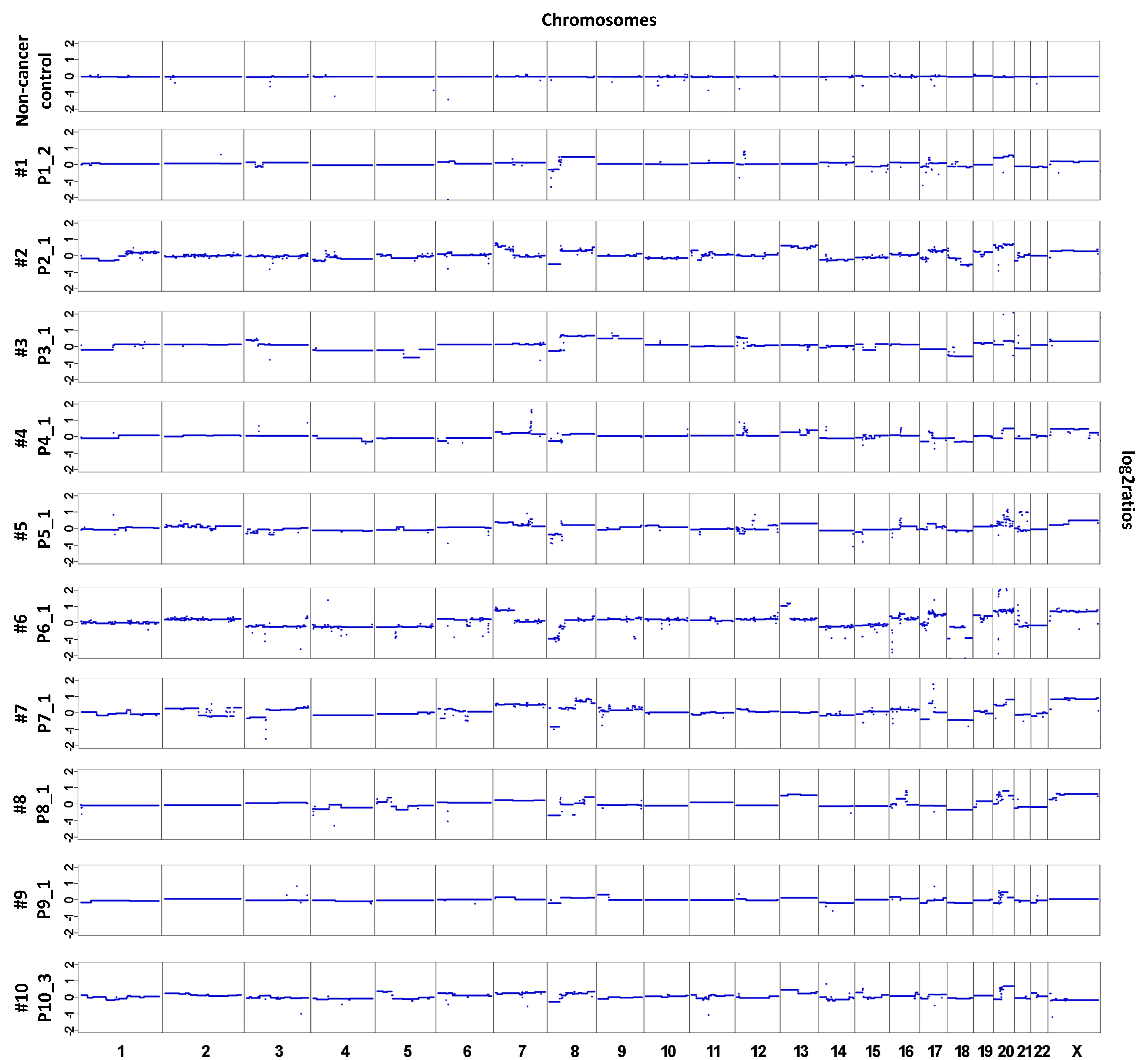

Supplement: Figure S1 — Plasma-Seq copy number profiles of a control, i.e. from a male person without cancer (shown on top; non-cancer control), and one representative copy number profile from each patient (the left column shows the patient id, the right column the sample id). The X- and Y-axes indicate the chromosome and the log2-ratios, respectively. (TIF) [file pgen.1004271.s001.tif]

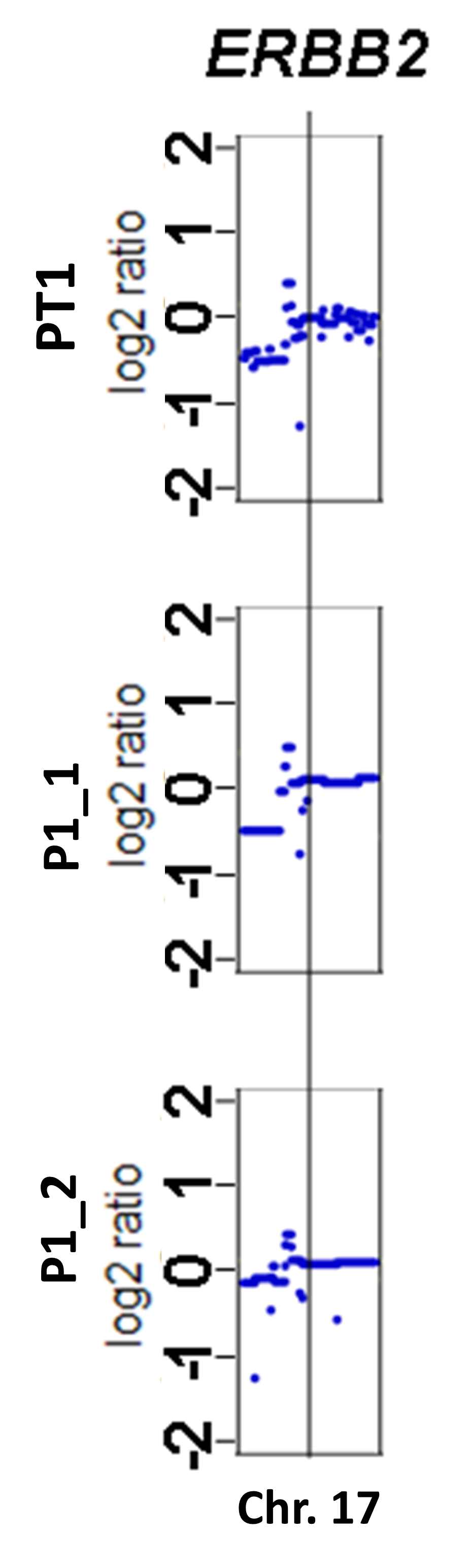

Supplement: Figure S2 — Log2-ratio blots of chromosome 17 from PT1, P1_1, and P1_2, demonstrating a focal amplification close to the centromere on 17q11.2 (chr17:26,205,340–29,704,695). The localization of the ERBB2 gene (chr17q12:37,844,167–37,886,679) is indicated by the black line. (TIF) [file pgen.1004271.s002.tif]

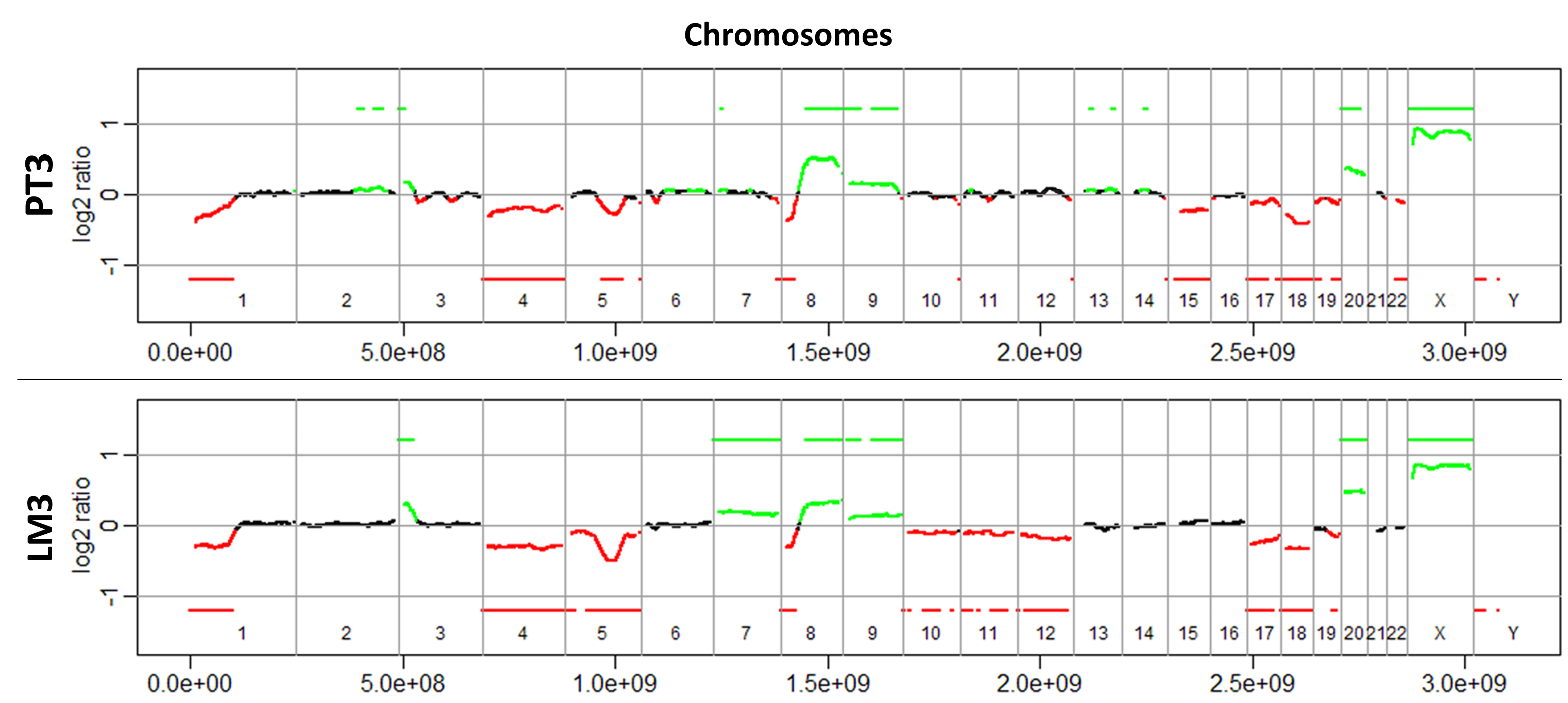

Supplement: Figure S3 — Two pre-treatment samples, i.e. primary tumor (PT3) and a liver metastasis (LM3), analyzed by array-CGH shared many copy number changes with the exception of chromosome 12, which was balanced in the primary but lost in the metastasis (red: lost regions; green: gained regions; black: balanced regions). (TIF) [file pgen.1004271.s003.tif]

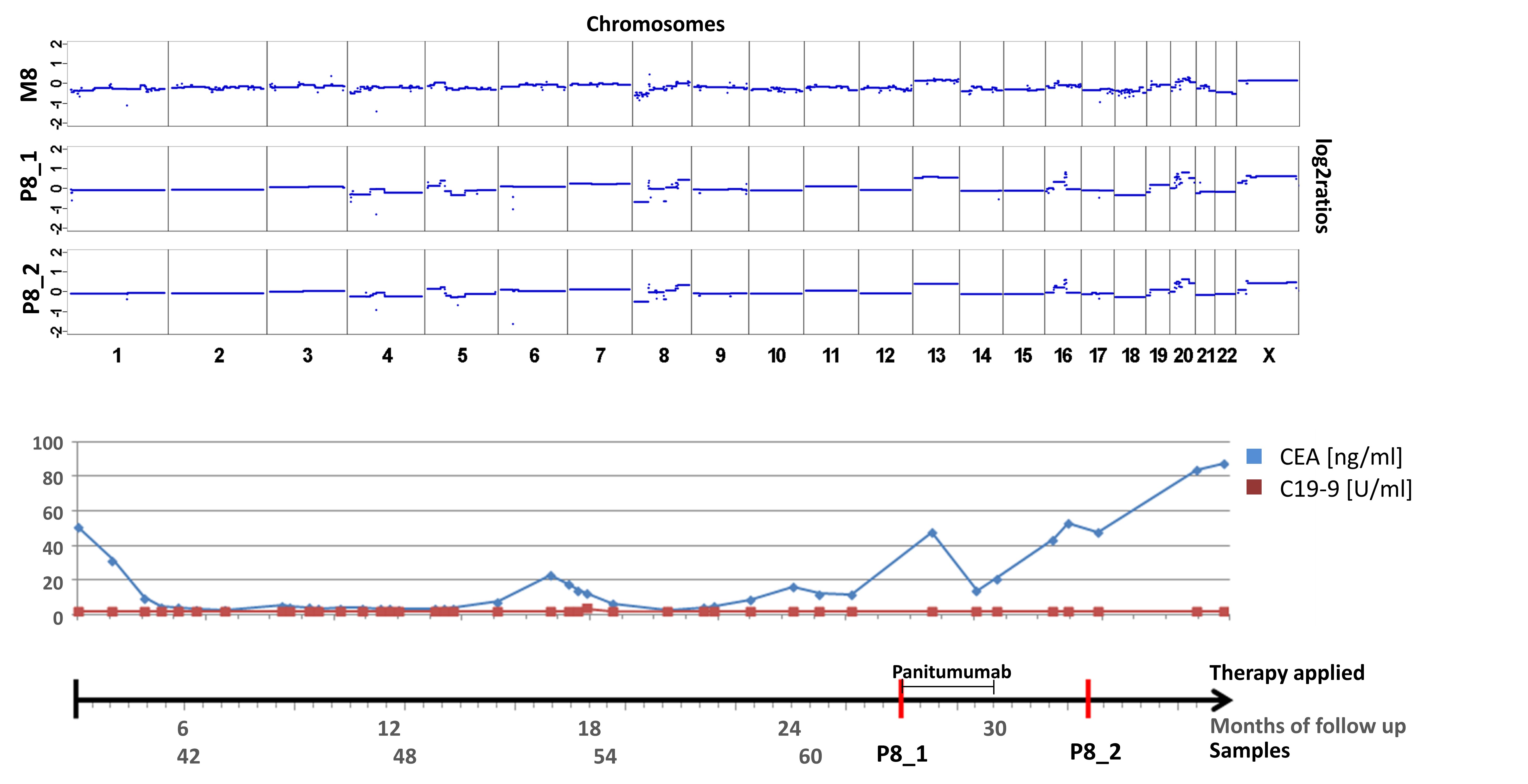

Supplement: Figure S4 — Analyses of three different samples from patient #8: Plasma-Seq profiles of a metastasis (M8) obtained 5 months after initial diagnosis, and pre-and post-treatment plasma-samples (P8_1 and P8_2, respectively). He had a brief response to panitumumab treatment, but relapsed within 4 months of treatment (radiological progression and increasing tumor markers). A second blood sample 10 months (P8_2) later demonstrated an almost identical copy number profile despite the 10 months' time interval between the 1st and the 2nd sample and despite the progressive disease (for further details see text). (TIF) [file pgen.1004271.s004.tif]

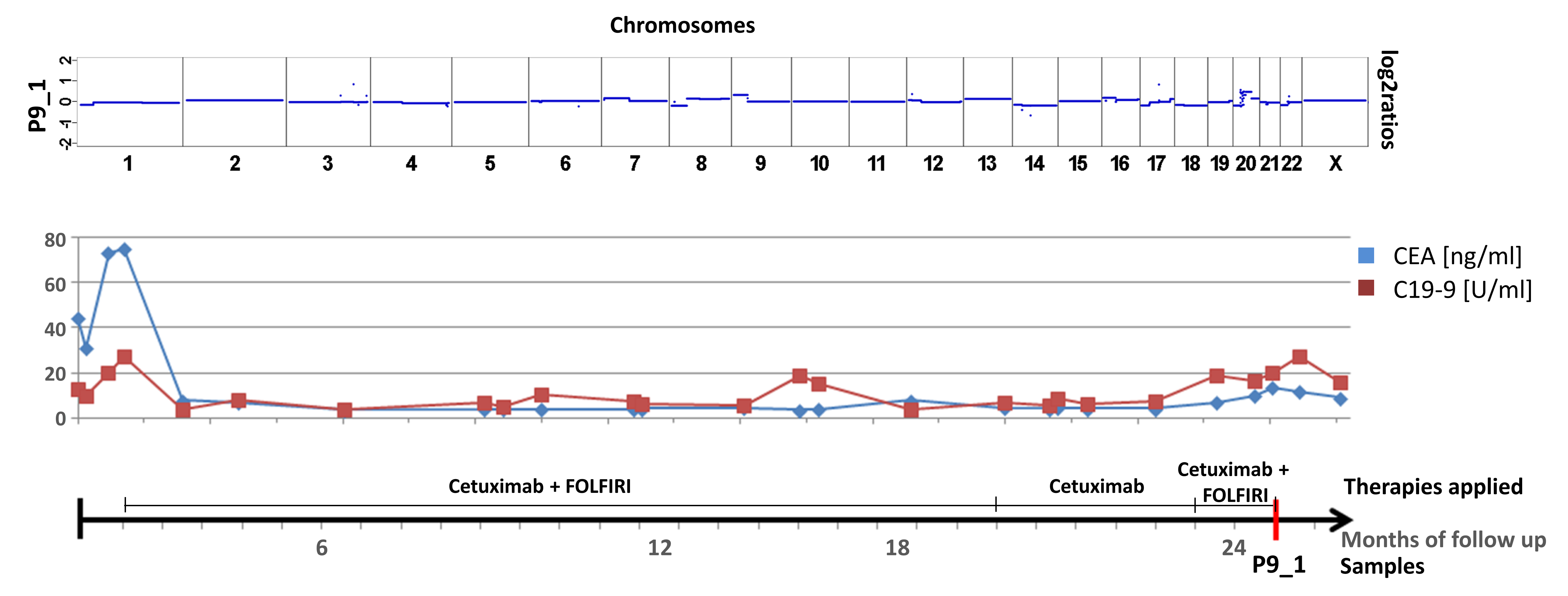

Supplement: Figure S5 — Patient #9 responded to cetuximab for more than 2 ½ years. After this long period of time a mild progress and mild increase of CEA and CA 19-9 were noted (CEA in blue and CA 19-9 in red). Plasma-Seq (P9_1) identified only few copy number changes, consistent with a low ctDNA fraction. (FOLFIRI: FOL-Folinic acid (leucovorin) + F-Fluorouracil (5-FU) + IRI-irinotecan). (TIF) [file pgen.1004271.s005.tif]

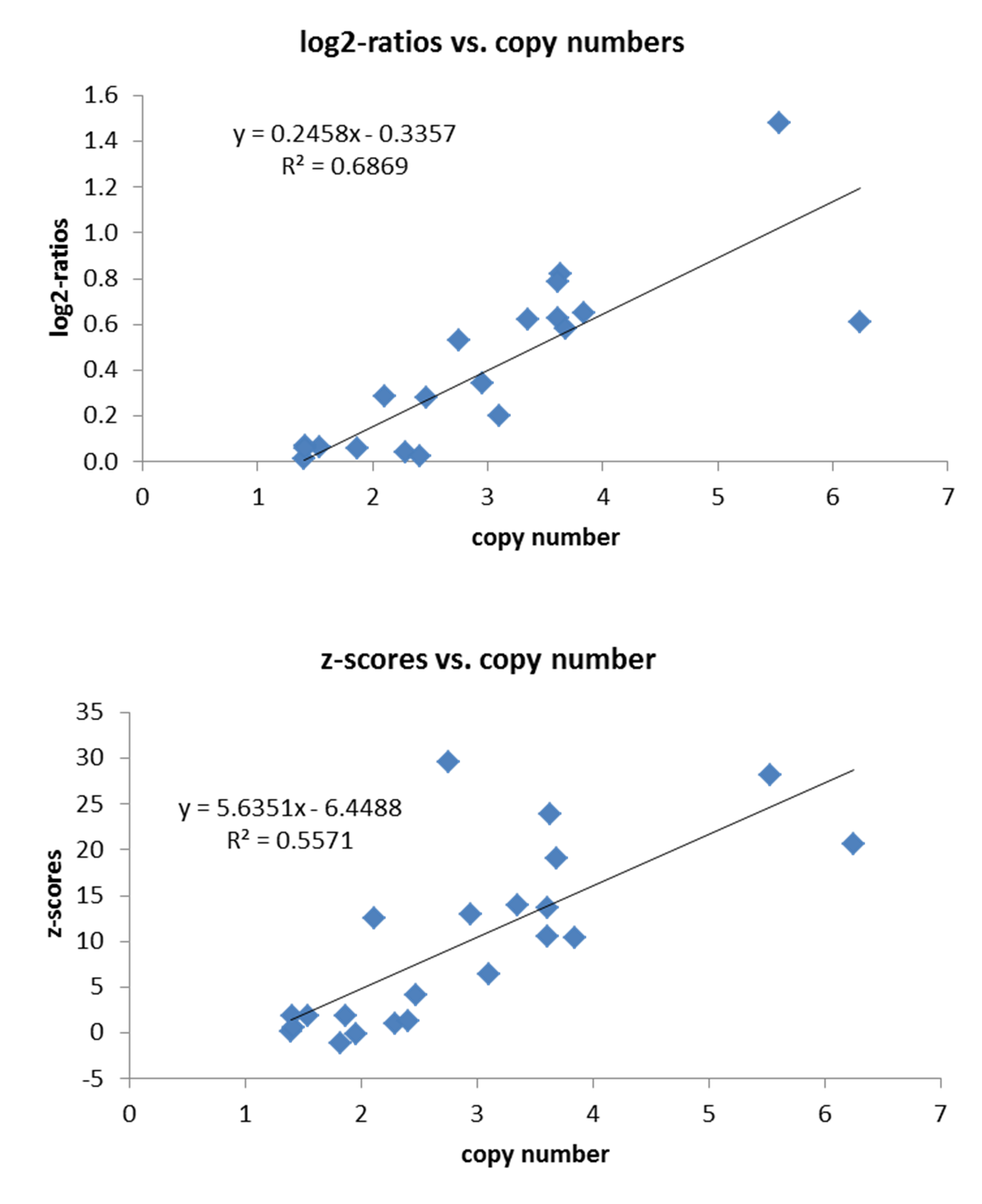

Supplement: Figure S6 — Validation of the KRAS, MET, and ERBB2 results established by plasma-Seq with TaqMan Copy Number assays. The graphs illustrate on the X-axes the relative copy numbers as established by the TaqMan assays and the Y-axes indicate the log2-ratios and the z-scores, respectively. (TIF) [file pgen.1004271.s006.tif]
