# Supplementary material for: Changes in Colorectal Carcinoma Genomes under Anti-EGFR Therapy Identified by Whole-Genome Plasma DNA Sequencing
Source: PLoS Genet. 2014 Mar 27;10(3):e1004271. doi: 10.1371/journal.pgen.1004271 (PMC3967949; doi:10.1371/journal.pgen.1004271)
Supplement: Table S1 — Results of deep sequencing: The columns display the sample numbers and results for KRAS (codons 12 and 13), the BRAF V600E mutation, PIK3CA (exon-9 and exon-20), and for the EGFR S492R mutation. (DOCX) [file pgen.1004271.s007.docx]

**Table S1** Results of deep sequencing.

| **Sample** | ***KRAS* G12, G13** | | ***BRAF* V600E** | | ***PIK3CA* E542, E545** | | ***PIK3CA* H1047R** | | ***EGFR* S492R** | |
| --- | --- | --- | --- | --- | --- | --- | --- | --- | --- | --- |
|  | **Reads** | **% mutated** | **Reads** | **% mutated** | **Reads** | **% mutated** | **Reads** | **% mutated** | **Reads** | **% mutated** |
| **P1_1** | 90007 | 0 | 19293 | 0 | 129371 | 0 | 106907 | 0 | Not done | |
| **P1_2** | 217644 | 0 | 20124 | 0 | 28832 | 0 | 462678 | 0 | Not done | |
| **P1_3** | 270058 | 0 | 16052 | 0 | 36172 | 0 | 150186 | 0 | Not done | |
| **PT1** | NA | | 785623 | 0 | 172759 | 0 | 303705 | 0 | Not done | |
| **P2_1** | 89082 | 0 | 17774 | 0 | 416409 | 6 (E545K) | 148531 | 0 | Not done | |
| **P2_2** | Not analyzable | | 173792 | 0 | 570919 | 2 (E545K) | 221193 | 0 | Not done | |
| **PT2** | 158225 | 0 | 611906 | 0 | 193593 | 16 (E545K) | 271974 | 0 | Not done | |
| **P3_1** | 214266 | 0 | 209166 | 0 | 155205 | 0 | 170213 | 0 | 103027 | 0 |
| **PT3** | Not analyzable | | 138943 | 0 | 126095 | 0 | 153297 | 0 | 66259 | 0 |
| **P4_1** | 172400 | 0 | 504957 | 0 | 114919 | 0 | 478558 | 0 | Not done | |
| **PT4** | No material left | | 457411 | 0 | No material left | | No material left | | Not done | |
| **P5_1** | 404816 | 0 | 1035911 | 0 | 81270 | 1 (E545A) | 148609 | 0 | Not done | |
| **PT5** | Not analyzable | | 243214 | 0 | 135608 | 34 (E545A) | 232795 | 0 | Not done | |
| **P6_1** | 1107604 | 0 | 18490 | 0 | 158716 | 0 | 252822 | 0 | 307402 | 0 |
| **P6_2** | 596142 | 0 | 13031 | 0 | 196231 | 0 | 1272153 | 0 | 311872 | 0 |
| **P7_1** | No material left | | No material left | | No material left | | No material left | | Not done | |
| **P8_1** | 1247075 | 0 | 21560 | 0 | 267452 | 12 (E545A) | 123244 | 0 | Not done | |
| **P8_2** | 125748 | 0 | 523334 | 0 | 201813 | 0 | 925705 | 0 | Not done | |
| **PT8** | Not analyzable | | 376070 | 0 | Not analyzable | | 250316 | 0 | Not done | |
| **M8** | 92179 | 0 | 978065 | 0 | 282466 | 2 (E545A) | 370941 | 0 | Not done | |
| **P9_1** | 633231 | 0 | 15893 | 0 | 655031 | 0 | 144973 | 0 | 544936 | 0 |
| **P10_1** | 187659 | 0 | 481951 | 0 | 153076 | 0 | 312246 | 0 | Not done | |
| **P10_2** | 137591 | 0 | 781122 | 0 | 356943 | 0 | 356115 | 0 | Not done | |
| **P10_3** | 203474 | 0 | 260812 | 0 | 174585 | 0 | 222426 | 0 | Not done | |
| **C10** | 160940 | 38 (45)* | Not done | | Not done | | Not done | | Not done | |
| **C25** | 293712 | 9 (12)* | Not done | | Not done | | Not done | | Not done | |

* numbers in parentheses denote percentage of mutated alleles as determined previously by 454 FLX deep sequencing
