# Supplementary material for: Changes in Colorectal Carcinoma Genomes under Anti-EGFR Therapy Identified by Whole-Genome Plasma DNA Sequencing
Source: PLoS Genet. 2014 Mar 27;10(3):e1004271. doi: 10.1371/journal.pgen.1004271 (PMC3967949; doi:10.1371/journal.pgen.1004271)
Supplement: Table S2 — Genome-wide coverage of plasma and tumor samples after plasma-Seq. (DOCX) [file pgen.1004271.s008.docx]

**Table S2** Genome-wide coverage of plasma and tumor samples after plasma-Seq.

| **Samples** | **Total reads^1^** | **Median read-count per bin** | **Genome coverage** |
| --- | --- | --- | --- |
| **PT1** | 3381986 | 65 | 0.16 |
| **P1_1** | 2946103 | 55 | 0.14 |
| **P1_2** | 1557702 | 30 | 0.08 |
| **P1_3** | 1789996 | 35 | 0.09 |
| **PT2** | 305199 | 6 | 0.01 |
| **P2_1** | 3709161 | 69 | 0.18 |
| **P2_2** | 3436778 | 66 | 0.17 |
| **P2_3** | 8560681 | 166 | 0.41 |
| **P3_1** | 3071067 | 59 | 0.15 |
| **PT4** | 1625873 | 30 | 0.08 |
| **P4_1** | 8605534 | 165 | 0.42 |
| **PT5** | 3388012 | 63 | 0.16 |
| **P5_1** | 3444476 | 66 | 0.17 |
| **P6_1** | 3674579 | 67 | 0.18 |
| **P7_1** | 5762287 | 101 | 0.28 |
| **P8_1** | 1231270 | 23 | 0.06 |
| **P8_2** | 1419947 | 27 | 0.07 |
| **P9_1** | 3957753 | 77 | 0.19 |
| **P10_3** | 2451599 | 47 | 0.12 |

^1^ Total number of unique reads that were uniquely aligned to the genome
