# Supplementary material for: Changes in Colorectal Carcinoma Genomes under Anti-EGFR Therapy Identified by Whole-Genome Plasma DNA Sequencing
Source: PLoS Genet. 2014 Mar 27;10(3):e1004271. doi: 10.1371/journal.pgen.1004271 (PMC3967949; doi:10.1371/journal.pgen.1004271)
Supplement: Table S3 — Details of copy-number aberrant segments detected by plasma-Seq. (DOCX) [file pgen.1004271.s009.docx]

**Table S3** Details of copy-number aberrant segments detected by plasma-Seq.

|  | ***KRAS*** | | | | | | |
| --- | --- | --- | --- | --- | --- | --- | --- |
| **Sample** | **Chr** | **Start** | **End** | **Reads in segment^1^** | **Log2-ratio^2^** | **Relative copy-number^3^** | **Z-score^4^** |
| **P1_2** | chr12 | 24478385 | 26907992 | 2331 | 0.82 | 3.53 | 23.9 |
| **P1_3** | chr12 | 25211574 | 26625084 | 1277 | 0.62 | 3.07 | 13.9 |
| **P2_3** | chr12 | 25267911 | 25436943 | 928 | 0.79 | 3.46 | 10.59 |
| **P3_1** | chr12 | 23293114 | 34604925 | 15744 | 0.52 | 2.87 | 28.99 |
| **P4_1** | chr12 | 25324271 | 26342357 | 4803 | 0.63 | 3.10 | 13.63 |
| **P7_1** | chr12 | 16436904 | 29565516 | 23860 | 0.21 | 2.31 | 7.3 |
|  | ***MET*** | | | | | | |
| **Sample** | **Chr** | **Start** | **End** | **Reads in segment^1^** | **Log2-ratio^2^** | **Relative copy-number^3^** | **Z-score^4^** |
| **P4_1** | chr7 | 116259840 | 116543892 | 2258 | 1.48 | 5.58 | 28.13 |
| **PT5** | chr7 | 112290085 | 118409416 | 12026 | 0.61 | 3.05 | 20.58 |
| **P5_1** | chr7 | 112007859 | 118409416 | 10539 | 0.58 | 2.99 | 19 |
| **P7_1** | chr7 | 102486040 | 127171171 | 53558 | 0.46 | 2.75 | 19.34 |
| **P8_1** | chr7 | 57609973 | 124234096 | 31851 | 0.23 | 2.35 | 7.65 |
| **P8_2** | chr7 | 0 | 170939734 | 86034 | 0.13 | 2.19 | 4.73 |
|  | ***ERBB2*** | | | | | | |
| **Sample** | **Chr** | **Start** | **End** | **Reads in segment^1^** | **Log2-ratio^2^** | **Relative copy-number^3^** | **Z-score^4^** |
| **PT2** | chr17 | 33159345 | 49412451 | 2468 | 0.34 | 2.53 | 6.69 |
| **P2_1** | chr17 | 36499046 | 38812163 | 4324 | 0.39 | 2.62 | 5.61 |
| **PT5** | chr17 | 33215703 | 48678979 | 22313 | 0.41 | 2.66 | 5.66 |
| **P5_1** | chr17 | 22154521 | 49808625 | 37834 | 0.3 | 2.46 | 5.01 |
| **P6_1** | chr17 | 33895217 | 38078885 | 9160 | 0.65 | 3.14 | 10.4 |
| **P7_1** | chr17 | 37402243 | 37966170 | 19029 | 4.2 | 36.76 | 196.4 |
|  | ***EGFR*** | | | | | | |
| **Sample** | **Chr** | **Start** | **End** | **Reads in segment^1^** | **Log2-ratio^2^** | **Relative copy-number^3^** | **Z-score^4^** |
| **PT2** | chr7 | 33305557 | 58029586 | 3117 | 0.21 | 2.31 | 7.79 |
| **P2_1** | chr7 | 50185479 | 57215018 | 11426 | 0.41 | 2.66 | 10.85 |
| **P2_3** | chr7 | 32561950 | 57734154 | 86393 | 0.2 | 2.30 | 4.96 |
| **PT5** | chr7 | 20838930 | 61520887 | 61690 | 0.41 | 2.66 | 11.98 |
| **P5_1** | chr7 | 41577059 | 57609972 | 24576 | 0.39 | 2.62 | 10.45 |
| **P6_1** | chr7 | 45256724 | 62018114 | 33801 | 0.78 | 3.43 | 11.7 |
| **P7_1** | chr7 | 45144040 | 55322256 | 25477 | 0.55 | 2.93 | 17.47 |
| **P8_1** | chr7 | 0 | 57609972 | 29034 | 0.25 | 2.38 | 8.71 |
| **P8_2** | chr7 | 0 | 170939734 | 86034 | 0.13 | 2.19 | 4.73 |

^1^ Raw read-counts of segmented bins including *KRAS, EGFR, ERBB2*, and *MET* identified by combined CBS and GLAD outputs from CGHweb package

^2^ Mean log2-ratios for each identified segment

^3^ Relative copy-number of aberrant segment. Calculated from log2 ratios by following formula: relative copy-number = 2*2^(log2-ratio). This does not reflect actual copy-number, since tumor-content is not known.

^4^ Z-scores for each identified segment
