# Supplementary material for: Changes in Colorectal Carcinoma Genomes under Anti-EGFR Therapy Identified by Whole-Genome Plasma DNA Sequencing
Source: PLoS Genet. 2014 Mar 27;10(3):e1004271. doi: 10.1371/journal.pgen.1004271 (PMC3967949; doi:10.1371/journal.pgen.1004271)
Supplement: Table S4 — Summary of copy number changes of KRAS, EGFR, ERBB2, and MET. (DOCX) [file pgen.1004271.s010.docx]

| **Patient** | **Gene** | **Primary Tumor** | **Pre-treatment** | **Post-treatment 1** | **Post-treatment 2** |
| --- | --- | --- | --- | --- | --- |
| **#1** | *KRAS* | - | - | focal amp | focal amp |
|  | *EGFR* | - | - | - | - |
|  | *ERBB2* | - | - | - | - |
|  | *MET* | - | - | - | - |
| **#2** | *KRAS* | - | - | - | focal amp |
|  | *EGFR* | polysomy 7p | polysomy 7p | - ^1^ | polysomy 7p |
|  | *ERBB2* | polysomy 17 | polysomy 17 | - ^1^ | - ^1^ |
|  | *MET* | - | - | - | - |
| **#3** | *KRAS* | - | n/a | polysomy 12p | n/a |
|  | *EGFR* | - | n/a | - | n/a |
|  | *ERBB2* | - | n/a | - | n/a |
|  | *MET* | - | n/a | - | n/a |
| **#4** | *KRAS* | n/a^2^ | n/a | focal amp | n/a |
|  | *EGFR* | n/a^2^ | n/a | - | n/a |
|  | *ERBB2* | n/a^2^ | n/a | - | n/a |
|  | *MET* | n/a^2^ | n/a | focal amp | n/a |
| **#5** | *KRAS* | - | n/a | - | n/a |
|  | *EGFR* | polysomy 7p | n/a | polysomy 7p | n/a |
|  | *ERBB2* | amp | n/a | amp | n/a |
|  | *MET* | focal amp | n/a | focal amp | n/a |
| **#6** | *KRAS* | n/a | - | n/a^3^ | n/a |
|  | *EGFR* | n/a | polysomy 7p | n/a^3^ | n/a |
|  | *ERBB2* | n/a | polysomy 17q | n/a^3^ | n/a |
|  | *MET* | n/a | - | n/a^3^ | n/a |
| **#7** | *KRAS* | n/a^4^ | n/a | polysomy 12p | n/a |
|  | *EGFR* | n/a^4^ | n/a | polysomy 7 | n/a |
|  | *ERBB2* | amp^4^ | n/a | focal amp | n/a |
|  | *MET* | n/a^4^ | n/a | polysomy 7 | n/a |
| **#8** | *KRAS* | - ^5^ | - | - | n/a |
|  | *EGFR* | - ^5^ | polysomy 7 | polysomy 7 | n/a |
|  | *ERBB2* | - ^5^ | - | - | n/a |
|  | *MET* | - ^5^ | polysomy 7 | polysomy 7 | n/a |
| **#9** | *KRAS* | n/a | n/a | - | n/a |
|  | *EGFR* | n/a | n/a | - | n/a |
|  | *ERBB2* | n/a | n/a | - | n/a |
|  | *MET* | n/a | n/a | - | n/a |
| **#10** | *KRAS* | n/a | - | - | - |
|  | *EGFR* | n/a | - | - | - |
|  | *ERBB2* | n/a | - | - | - |
|  | *MET* | n/a | - | - | - |

**Table S4** Summary of copy number changes of *KRAS, EGFR, ERBB2*, and *MET.*

^1^ Polysomy was not detected owing to a low amount of tumor DNA

^2^ Only a biopsy was available that may not represent genetic composition of primary

^3^ Amount of tumor DNA in samples was too low to obtain tumor-specific copy number aberrations

^4^ Only biopsy was available, *ERBB2* amplification based on immunohistochemistry and silver *in situ* hybridization

^5^ Quality of plasma-Seq data did not allow reliable z-score calculation
